# Genetic incorporation of the protein transduction domain of Tat into Ad5 fiber enhances gene transfer efficacy

**DOI:** 10.1186/1743-422X-4-103

**Published:** 2007-10-24

**Authors:** Tie Han, Yizhe Tang, Hideyo Ugai, Leslie E Perry, Gene P Siegal, Juan L Contreras, Hongju Wu

**Affiliations:** 1 Division of Human Gene Therapy, Department of Medicine, University of Alabama at Birmingham, Birmingham, USA; 2 Division of Human Gene Therapy, Departments of Pathology, University of Alabama at Birmingham, Birmingham, USA; 3 Division of Human Gene Therapy, Departments of Surgery, University of Alabama at Birmingham, Birmingham, USA; 4 Division of Human Gene Therapy, Departments of Cell Biology, University of Alabama at Birmingham, Birmingham, USA; 5 Division of Human Gene Therapy, Departments of Obstetrics and Gynecology, University of Alabama at Birmingham, Birmingham, USA; 6 Gene Therapy Center, University of Alabama at Birmingham, Birmingham, USA

## Abstract

**Background:**

Human adenovirus serotype 5 (Ad5) has been widely explored as a gene delivery vector for a variety of diseases. Many target cells, however, express low levels of Ad5 native receptor, the Coxsackie-Adenovirus Receptor (CAR), and thus are resistant to Ad5 infection. The Protein Transduction Domain of the HIV Tat protein, namely PTD_tat_, has been shown to mediate protein transduction in a wide range of cells. We hypothesize that re-targeting Ad5 vector via the PTD_tat _motif would improve the efficacy of Ad5-mediated gene delivery.

**Results:**

In this study, we genetically incorporated the PTD_tat _motif into the knob domain of Ad5 fiber, and rescued the resultant viral vector, Ad5.PTD_tat_. Our data showed the modification did not interfere with Ad5 binding to its native receptor CAR, suggesting Ad5 infection via the CAR pathway is retained. In addition, we found that Ad5.PTD_tat _exhibited enhanced gene transfer efficacy in all of the cell lines that we have tested, which included both low-CAR and high-CAR decorated cells. Competitive inhibition assays suggested the enhanced infectivity of Ad5.PTD_tat _was mediated by binding of the positively charged PTD_tat _peptide to the negatively charged epitopes on the cells' surface. Furthermore, we investigated *in vivo *gene delivery efficacy of Ad5.PTD_tat _using subcutaneous tumor models established with U118MG glioma cells, and found that Ad5.PTD_tat _exhibited enhanced gene transfer efficacy compared to unmodified Ad5 vector as analyzed by a non-invasive fluorescence imaging technique.

**Conclusion:**

Genetic incorporation of the PTD_tat _motif into Ad5 fiber allowed Ad5 vectors to infect cells via an alternative PTD_tat _targeting motif while retaining the native CAR-mediated infection pathway. The enhanced infectivity was demonstrated in both cultured cells and in *in vivo *tumor models. Taken together, our study identifies a novel tropism expanded Ad5 vector that may be useful for clinical gene therapy applications.

## Background

Human adenovirus serotype 5 (Ad5) has been widely exploited as a gene delivery vector, owing largely to its superior gene delivery efficacy, minor pathological effect on humans, and easy manipulation *in vitro*. Several problems, however, have been identified in the course of development and application of Ad5-based gene therapy protocols, one of which is the inefficient gene delivery into target cells [1-3]. It is known that infection of Ad5 is initiated by attachment of its capsid fiber protein to the cell surface coxsackievirus adenovirus receptor (CAR), which is followed by interaction of its penton base with α_v _integrins that triggers the internalization of the viruses [4-7]. Many target cells, such as malignant tumor cells, are found to express very low level of CAR, and thus are resistant to Ad5 infection. Therefore, strategies to re-direct Ad5 infection via alternative receptors would be useful for gene therapy applications.

Since fiber, the capsid protein extruding from the Ad virion surface, is an essential mediator of Ad5 infection, fiber modification has been explored as a means to re-direct Ad5 tropism [1]. Ad5 fiber is composed of an N-terminal tail that is attached to a penton base on the virion surface, a shaft domain consisting of 22 repeats of a 15-amino acid residue motif, and a C-terminal globular domain, named knob, which functions as a receptor binding domain. Because of the essential role of the fiber knob domain in mediating Ad5 infection, knob modification could be one of the most effective ways to re-direct Ad5 tropism. Indeed, both genetic and non-genetic strategies have been shown to successfully retarget Ad5 vectors. For example, bi-specific adapter proteins that bind both the knob domain and an alternative receptor expressed on the surface of the target cells have been employed to re-direct Ad5 infection [8-11]. In addition, genetic incorporation of RGD peptide and/or a polylysine epitope into the knob domain allowed Ad5 to infect cells through alternative receptors (cell surface integrins for RGD and negatively charged epitopes such as heparan sulfate proteoglycans for polylysine), thus greatly improving the gene delivery efficacy Ad5 vectors in many target cells [12-15].

Protein transduction domains (PTD) or Cell Penetrating Peptides (CPP) are a class of small peptides that can traverse the plasma membrane of many, if not all, mammalian cells [16-20]. Among these peptides, the PTD of the Tat protein (PTD_tat_) of human immunodeficiency viruses types 1 and 2 (HIV-1 and HIV-2) has been one of the most widely studied PTDs. PTD_tat _consists of 11 highly basic amino acid residues, YGRKKRRQRRR [21,22]. The mechanism of how PTD_tat _crosses the cell membrane has been intensively studied, but controversies remain [23-26]. Nonetheless, it is commonly agreed upon that the interaction between the positive charge of the PTD domain and the negative epitopes, in particular, the heparan sulfate proteoglycans expressed on cell membranes, plays an essential role in the internalization of PTD_tat _fusion proteins [17,20,27]. Further studies suggest that the interaction between PTD_tat _and heparan sulfate is specified by both charge and structure of the peptide and the proteoglycans [17,27-30].

Given the potential importance of the PTDs in drug delivery, much interest has been generated in exploiting this system as a tool to deliver therapeutic molecules or particles into mammalian cells. PTDs have already been widely used in the field of protein therapy whereby PTDs are fused to the protein of interest, and used to deliver the heterologous protein into cultured cells [17,20,31]. Interestingly, it has been demonstrated in several mouse studies that PTD_tat _fusion proteins can be delivered into different tissues *in vivo *following systemic administration, and therapeutic benefits have been observed [32-35]. In addition, PTDs have been used to deliver other large molecules or particles including plasmids, liposomes, nanoparticles, phages and viruses, with variable efficiency [36-41]. In these applications, PTDs were conjugated to the vehicle of interest by incubation in coupling solutions. In other words, the coating of the vehicle was not based on genetic modification, but on ionic or other interactions between the peptides and the vehicle.

Because of the potency of PTD_tat _in mediating cellular uptake of small and large molecules, in this study, we attempted to re-direct Ad5 infection via the PTD_tat _pathway. Previous studies have demonstrated pre-treatment of Ad particles with chemically synthesized PTDs or bi-specific adaptor proteins composed of the extracellular domain of CAR and PTDs improved Ad infection [37,42]. Nonetheless, intrinsic to these non-genetic modification strategies, the efficiency of retargeting depended on the affinity and stability of protein-protein interactions, and thus may be highly variable in different systems. In addition, a large amount of peptide or adaptor protein is seen to be required for *in vivo *investigations. Our study was designed to retarget Ad5 vectors to the PTD_tat _pathway using a genetic capsid modification strategy. We genetically incorporated the sequences encoding the PTD_tat _peptide into the 3' end of the Ad5 fiber gene, rescued the modified viruses, and characterized them in detail. Our data demonstrated that genetic modification of Ad5 fiber with the PTD_tat _motif greatly improved the efficacy of gene delivery in both cultured cells and in tumor models. Our study thus identified a novel tropism expanded Ad5 vector that may be useful for clinical gene therapy applications, especially for applications involving gene delivery into low-CAR expressing cells.

## Results

### Development of PTD_tat_-modified Ad5 vector – Ad5.PTD_tat_

**Figure 1:**
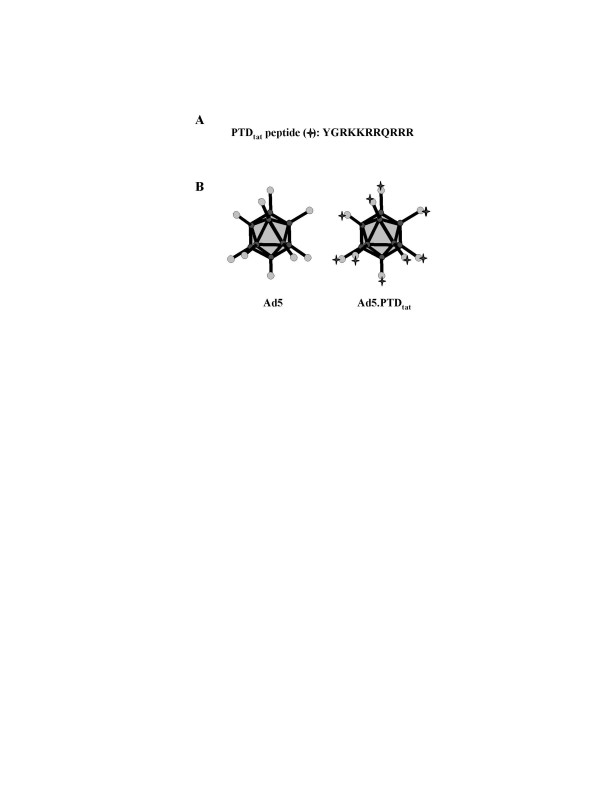
**Diagram of PTD_tat _modified Ad5 vector**. (A) PTD_tat _peptide incorporated into the fiber knob domain. (B) Structural diagram of Ad5 and Ad5.PTD_tat _vector. The PTD_tat _motif was incorporated at the C-terminal end of the fiber.

As the receptor binding domain, the knob of the Ad5 fiber has been shown to be an effective site for incorporating foreign targeting motifs [12-15]. In this study, we genetically incorporated the PTD_tat _epitope into the C-terminal end of the fiber knob domain (Fig. 1). The Ad5 genome contains about 36 kilobases (kb) and is too large for direct modification using conventional cloning techniques. To achieve our goal, we therefore established a bacteria-based homologous recombination system for Ad5 fiber modification [15]. Using this system, the nucleotide sequences encoding PTD_tat _were incorporated into the 3'end of the fiber gene, immediately before the stop code. The modified Ad5 (Ad5.PTD_tat_) and the unmodified control (Ad5) were both replication deficient as their E1 region, which is essential for Ad5 replication, was replaced with a CMV promoter-driven green fluorescence protein (GFP) reporter gene. The viruses were rescued in 293 cells stably expressing Ad-E1 genes, and purified with CsCl gradient ultracentrifugation. The yield of Ad5.PTD_tat _total viral particles (VPs) and the ratio of VPs : plaque formation units (pfu) were in the same range as that of unmodified Ad5 viruses, suggesting that the modification did not interfere with virus formation (data not shown). The modification was confirmed by both polymerase chain reaction (PCR) and sequence analysis of the modified region of the viral genome using viral DNA from purified Ad5 and Ad5.PTD_tat _viruses (data not shown).

### CAR-binding activity of Ad5.PTD_tat_

**Figure 2:**
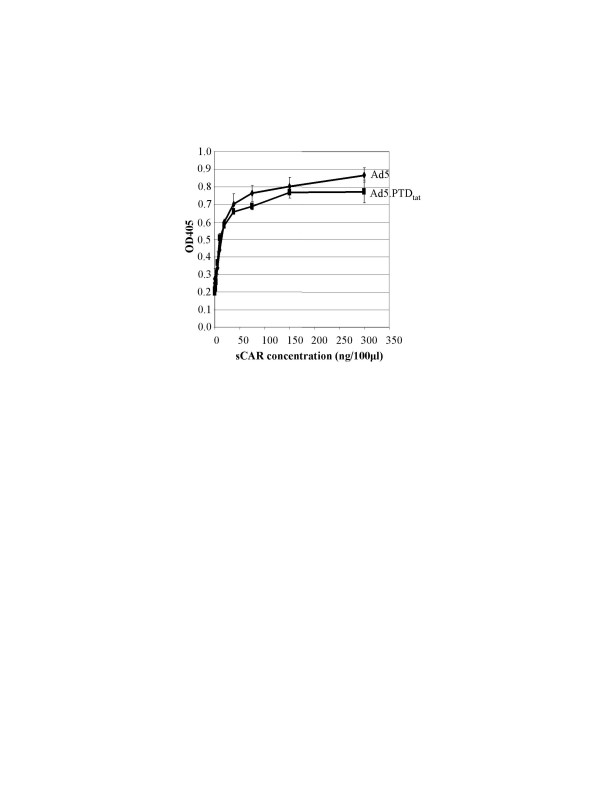
**Ad5.PTD_tat _showed similar CAR-binding activity to unmodified Ad5 vector in an ELISA-based binding assay**. In the experiment, 10^9 ^VPs of each viral vector were immobilized in the wells of a 96-well ELISA plate, and incubated with increasing concentrations of recombinant sCAR (extracellular domain of CAR, i.e. soluble CAR). The binding activity was detected by AP activity conjugated to detection antibodies.

Unmodified Ad5 viruses interact with their native receptor CAR via the fiber knob domain. We thus examined whether incorporation of PTD_tat _into the knob domain interfered with the Ad5-CAR interaction. An enzyme-linked immunosorbent assay (ELISA) was employed in this regard. In the assay, Ad5.PTD_tat _or Ad5 viral particles were immobilized in the wells of a 96-well maxi-sorp plate, and incubated with varying amounts of recombinant extracellular domain of CAR (sCAR) protein. After extensive washing, binding of sCAR to the viruses were assessed with an anti-CAR antibody and corresponding secondary antibody conjugated to alkaline phosphatase (AP). The OD405 readings resulting from the color reaction with an AP substrate correspond to the binding activity of sCAR to the viruses. As shown in Fig. 2, binding of sCAR to Ad5.PTD_tat _is similar to that of unmodified Ad5, suggesting the genetically modified vector Ad5.PTD_tat _maintained its ability to interact with the Ad5 native receptor, CAR.

### Cell-binding activities of Ad5.PTD_tat_

**Figure 3:**
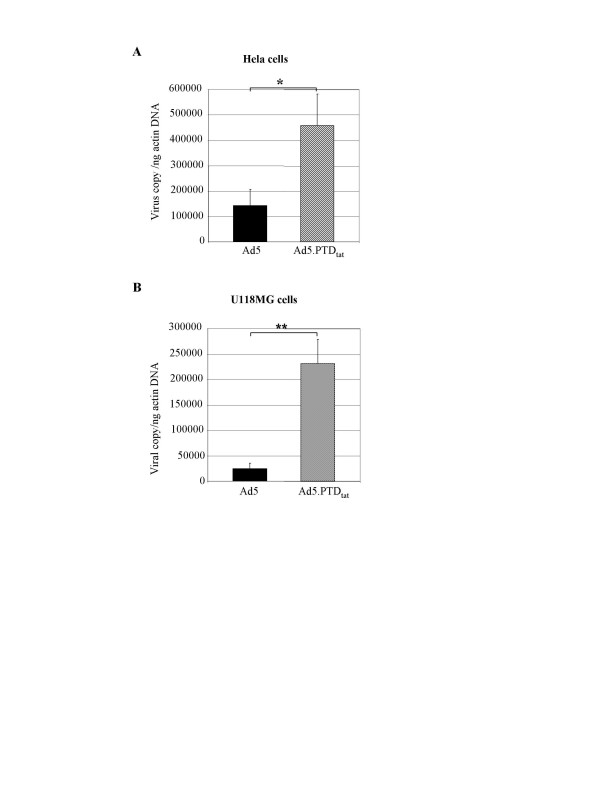
**PTD_tat _modification promoted Ad5 binding to cell surfaces**. Binding of Ad5 and Ad5.PTD_tat _were examined in both high-CAR expressing Hela cells (A) and low-CAR expressing U118MG cells (B) at 4°C. The amount of viruses associated with the cells was determined by quantitative PCR after DNA isolation from the cell lysate, and the viral copy numbers were normalized to actin DNA in the samples. The * indicates *p *< 0.05 and ** indicates *p *< 0.01 as analyzed by the *Student's t-test*.

The fiber knob domain of Ad is responsible for Ad5 binding to its target cells, which is the initial step in viral infection. Ad5.PTD_tat _was designed to re-direct Ad5 infection. We thus examined whether PTD_tat _modification had any effect on Ad5 binding to cells. To distinguish viruses bound to cells from viruses internalized into the cells, we performed a cell binding assay at 4°C since Ad internalization occurs through receptor-mediated endocytosis which is energy dependent, and is thus inhibited at 4°C [5,7]. In the assay, Ad5.PTD_tat _or control Ad5 was incubated with cells expressing different levels of CAR at 4°C for 1 hour, and the bound viral particles were examined by a quantitative PCR assay which assessed the viral genome copies in the cell lysates. We found that Ad5.PTD_tat _exhibited a significant higher cell-binding activity in almost all of the cells we examined, including both high-CAR and low-CAR containing cells. Shown in Fig. 3 are results obtained in two representative cell lines: high-CAR expressing Hela cells, and low-CAR expressing U118MG cells [43,44].

### Enhanced gene transfer efficacy of Ad5.PTD_tat_

**Figure 4:**
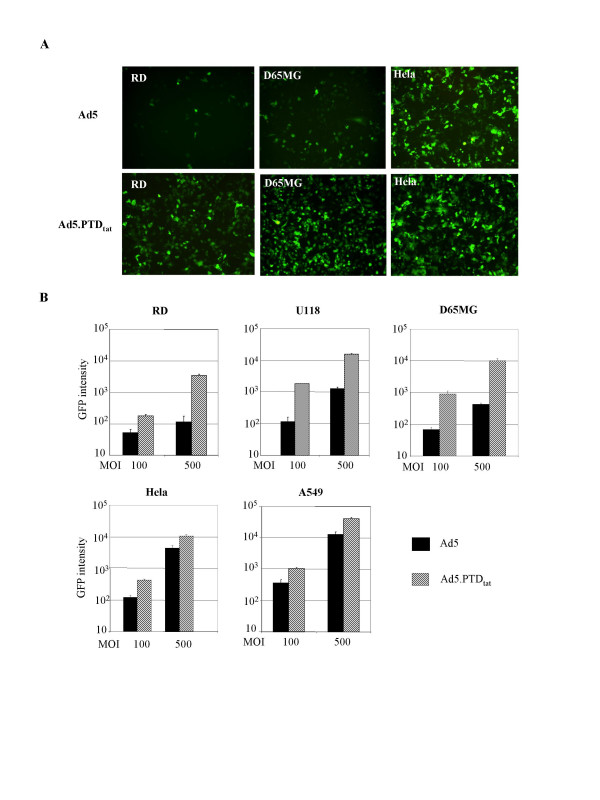
**Ad5.PTD_tat _exhibited enhanced gene transfer efficacy in a variety of tumor cells**. Gene transfer efficacy was evaluated by use of a GFP reporter that was carried in the E1 region of each vector. In the assay, tumor cells expressing varying levels of CAR were infected with either Ad5 or Ad5.PTD_tat _at an MOI of 100 or 500 VPs/cell, and GFP expression was examined by fluorescence microscopy and a fluorescence plate reader. (A) Representative fluorescence images of low-CAR containing cells (RD), medium-CAR containing cells (D65MG) and high-CAR expressing cells (Hela) that were infected with Ad5 or Ad5.PTD_tat _at an MOI of 500 VPs/cell. (B) GFP expression in a variety of cells infected with either Ad5 or Ad5.PTD_tat _was quantified using a fluorescence plate reader.

We further investigated the gene transfer efficacy of Ad5.PTD_tat _in a variety of cultured cells using the reporter GFP protein. Ad5.PTD_tat _vector or unmodified Ad5 was used to infect cells at different multiplicities of infection (MOIs). Two days after infection, we evaluated the transgene expression using a fluorescent microscope and a fluorescent plate reader. We found that Ad5.PTD_tat _showed more efficient gene delivery than unmodified Ad5 in all of the cells tested (Fig. 4). In particular, Ad5.PTD_tat _exhibited significantly higher gene transfer efficacy than unmodified Ad5 in the cells expressing low or medium levels of CAR such as RD cells, U118MG cells, and D65MG cells [43,44]. In high-CAR expressing cells that are readily accessible to unmodified Ad5 vector, Ad5.PTD_tat _also showed enhanced infectivity, presumably because Ad5.PTD_tat _maintained the CAR-mediated infection pathway while gaining extra targeting activity through the PTD_tat _pathway (Fig. 4).

### Identification of pathways mediating Ad5.PTD_tat _infection

**Figure 5:**
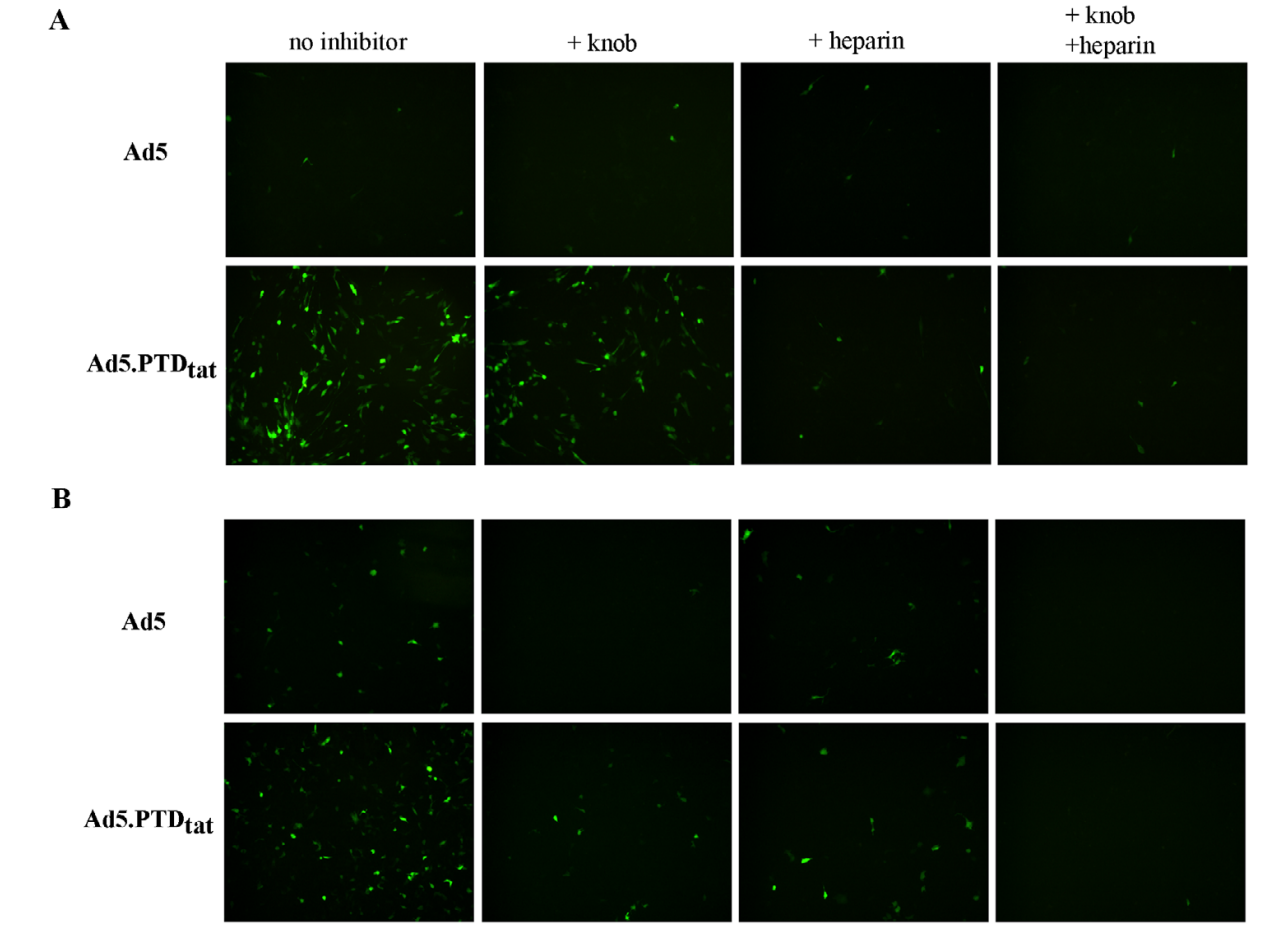
**Competitive inhibition assay showing the enhanced gene transfer efficacy of Ad5.PTD_tat _was mediated by the PTD_tat _motif**. In this assay, recombinant knob protein (50 μg/ml) was used to block CAR-mediated viral infection, and heparin (100 μg/ml) was used to block PTD_tat _mediated infection. Infections were performed at an MOI of 100 VPs/cell. (A) In low-CAR expressing U118MG cells that were resistant to unmodified Ad5 vector, Ad5.PTD_tat _mediated efficient gene delivery and the efficacy was completely inhibited by heparin, while recombinant knob had little effect, suggesting the enhanced infectivity of Ad5.PTD_tat _in low-CAR expressing cells resulted from the PTD_tat _motif. (B) In high-CAR expressing A549 cells, Ad5.PTD_tat _mediated gene delivery was partially inhibited with either knob or heparin, while being completely inhibited in the presence of both inhibitors, suggesting Ad5.PTD_tat _infected high-CAR expressing cells via both CAR and PTD_tat _pathways.

Ad5.PTD_tat _showed enhanced gene delivery efficacy compared to unmodified Ad5 vectors. To confirm that this expanded tropism was mediated by the genetically incorporated targeting motif PTD_tat_, we performed a gene transfer assay in the presence of competitive inhibitors. It has been shown that the interaction between the positively charged PTD_tat _and the negatively charged cell surface epitopes such as heparan sulfate proteoglycans is essential for PTD_tat _mediated protein transduction. Heparin, the structural analogue of heparan sulfate, would thus be expected to inhibit PTD_tat _mediated infection. In addition, recombinant knob protein was used to block the native CAR-mediated Ad5 infection because it compete with Ad5 vectors for cell surface CAR. In low-CAR containing U118MG cells [44], due to the paucity of CAR, unmodified Ad5 showed poor gene transfer efficacy, and neither knob nor heparin had any effect on Ad5-mediated transgene expression (Fig. 5A). In contrast, Ad5.PTD_tat _exhibited efficient gene delivery into U118MG cells, which was completely inhibited by heparin, but not by the recombinant knob protein (Fig. 5A). These data demonstrated Ad5.PTD_tat _infected low-CAR expressing cells mainly through the incorporated PTD_tat _motif. In high-CAR containing A549 cells [43], infection of unmodified Ad5 was completely blocked by recombinant knob protein while heparin had little effect, confirming that unmodified Ad5 mainly infected cells through the CAR pathway (Fig. 5B). On the other hand, Ad5.PTD_tat_-mediated gene transfer was partially blocked by either knob or heparin, but completely blocked in the presence of both knob and heparin, suggesting Ad5.PTD_tat _could infect cells via both CAR and the PTD_tat _motif (Fig. 5B).

### *In vivo *gene transfer efficacy of Ad5.PTD_tat_

**Figure 6:**
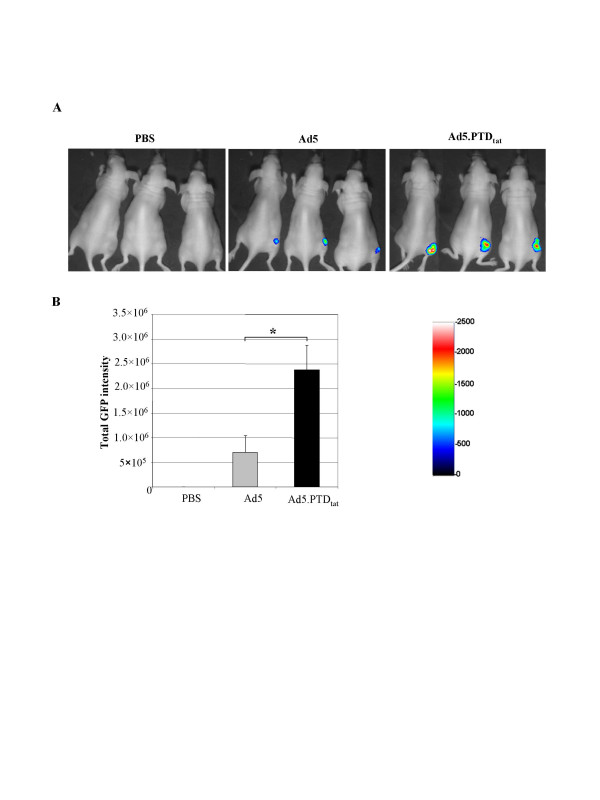
**PTD_tat _modification of Ad5 fiber enhanced *in vivo *gene delivery efficacy of the vector**. *In vivo *gene delivery of Ad5.PTD_tat _was examined using a non-invasive fluorescence imaging technique in low-CAR expressing tumor models. 10^10 ^VPs of Ad5 or Ad5.PTD_tat _were injected into the subcutaneous U118MG tumors, and *in vivo *green fluorescence images were acquired at different days post viral injection. (A) Representative *in vivo *images from PBS, Ad5, or Ad5.PTD_tat _injected mouse tumor models at day 7 after vector administration. The colors representing different intensities of signal are shown on the color bar. Ad5.PTD_tat _infection resulted in more intensive GFP signals than unmodified Ad5 vectors. (B) Quantitative analysis of the GFP intensity in the tumor model of each group. The * marks significant differences (p < 0.01) as analyzed by the *Student's t-test*.

We next examined whether the infectivity-enhanced vector Ad5.PTD_tat _could deliver enhanced gene transfer efficacy *in vivo*. Since Ad5.PTD_tat _showed more profound infectivity enhancement for low-CAR expressing tumor cells *in vitro*, we assessed the *in vivo *gene delivery efficacy of the Ad5 vectors using tumor models established with low-CAR containing U118MG cells. After the tumors were established subcutaneously in athymic (nude) mice, PBS, unmodified Ad5, or Ad5.PTD_tat _vectors were injected into the tumors. The gene delivery efficacy of each vector was analyzed by non-invasive fluorescence imaging that detected GFP expression in live mice. As shown in Fig. 6A, Ad5.PTD_tat_-infected tumors showed more intensive green fluorescence signals than Ad5-infected tumors, while no signal was detected in PBS-injected tumors. Quantitative analysis of the green fluorescence signals revealed that Ad5.PTD_tat_-mediated GFP expression was significantly higher than that of unmodified Ad5 vector in the tumors (*p *< 0.01) (Fig. 6B). These data suggest the infectivity-enhanced Ad5.PTD_tat _vector could be a useful vector for *in vivo *gene delivery into tumors, which is essential for cancer gene therapy.

## Discussion

In this study, we sought to improve the gene transfer efficacy of Ad 5 vectors by genetic modification of the fiber knob domain with a PTD_tat _motif. Our data demonstrated the success of this strategy. The fiber modified Ad5 vector, Ad5.PTD_tat_, not only exhibited enhanced gene delivery efficiency of Ad5 vectors in low-CAR cells that are resistant to unmodified Ad5 infection, but also in high-CAR cells that are permissive to Ad5 infection. The enhanced infectivity of Ad5.PTD_tat _was found to be mediated by targeting of PTD_tat _to the negatively charged epitopes such as heparan sulfate containing proteoglycans on cell surface. In addition, we found PTD_tat _mediated Ad5.PTD_tat _infection is additive to native CAR-mediated infection as assessed by competitive inhibition assays, which was not unexpected since Ad5.PTD_tat _maintained full CAR-binding activity. More significantly, the enhanced gene delivery efficacy of Ad5.PTD_tat _was demonstrated *in vivo *using low-CAR U118MG tumor models, and employment of a recently developed non-invasive optical imaging system allowed us to visually detect the enhanced gene delivery *in vivo*.

As a cell penetrating peptide, PTD_tat _is capable of traversing the plasma membrane of mammalian cells. Since the initial description that PTD_tat _is responsible for the ability of the HIV Tat protein to enter mammalian cells, PTD_tat _has attracted tremendous interest as a drug delivery vehicle [16-20]. Further interest has been stimulated by the observation that PTDs can facilitate systemic delivery of biologically active recombinant proteins *in vivo *[32-35,37]. Since inefficient gene delivery into target cells has been one of the major limitations in Ad5-mediated gene therapy, in this study, we attempted to employ PTD_tat _peptide to facilitate Ad5 mediated gene delivery. Employment of PTDs to facilitate virus infection has been investigated previously, but only using non-genetic methods [37,42]. In particular, chemically synthesized PTDs or bi-specific adaptor proteins consisting of PTDs and the extracelluar domain of CAR have been used to coat Ad vectors. These strategies too resulted in enhanced gene delivery [37,42]. Compared to the non-genetic methods, our genetically PTD_tat _modified vector has major advantages for two major reasons: 1) genetic modification allows stable interaction between Ad5 and the PTD_tat _targeting epitope, thus reducing the volatility associated with the affinity and stability of protein-protein interactions in the presence of different environmental factors. This is critical especially for *in vivo *applications; and 2) genetic modification does not require production of peptides or fusion proteins other than the viral vector, while large amounts of high quality protein/peptide production is required for non-genetic strategies (in addition to high quality production of the viral vectors), which is especially important for in vivo studies.

One issue associated with PTD_tat_-mediated protein delivery is the inefficient release of PTD_tat _fusion proteins from the endosomal compartments [24,45-48]. It has been demonstrated that a large proportion of the PTD_tat _fusion protein remains trapped in non-cytosolic compartments even though it is efficiently taken up by the cells. This apparently would compromise the therapeutic effect of the fusion protein. In our study, we examined the distribution of Ad5.PTD_tat _particles in cells at various time points (from 0.5 hour to 4 hours) following addition of the viruses to the cells by immunofluorescent staining, and found that the distribution of Ad5.PTD_tat _inside the cells was similar to that of unmodified Ad5 vectors (data not shown). This indicates endosomal trapping is not significant, if any present at all, with Ad5.PTD_tat _infection of cells. In addition, the enhanced gene delivery mediated by Ad5.PTD_tat _confirmed that the virions were able to efficiently escape the endosomal compartment.

The potential utility of the infectivity-enhanced Ad5.PTD_tat _vector in cancer gene therapy was initially investigated in this study using low-CAR expressing tumor models. Indeed, many tumor cells have been shown to express very low levels of CAR, which is partially responsible for the low efficacy of Ad5 mediated cancer gene therapy in *in vivo *studies, especially in clinical trials [1-3]. The ability of Ad5.PTD_tat _to improve the gene delivery efficacy is attributable to the PTD_tat _motif, which binds to the negatively charged motifs expressed on cell surface, in particular, heparan sulfate containing proteoglycans that are widely expressed in a variety of cells including tumor cells [49-51]. In addition to cancer gene therapy, Ad5.PTD_tat _may also be applied in other gene therapy applications where infectivity-enhancement is beneficial. Infectivity-enhanced vectors will not only allow efficient gene delivery into low-CAR target cells, but also allow use of a reduced amount of viral vectors, thus reducing vector-associated toxicity.

Previous studies have developed several other infectivity-enhanced vectors, which include Ad5 vectors modified with RGD, polylysine, or knobs from other Ad serotypes [13-15,52]. Since each of the modified vectors uses a unique extra targeting motif, the enhanced gene delivery efficacy in a specific cell type depends on the expression of individual receptors on its cell surface. Similar to PTD_tat_, the polylysine epitope, which is composed of a stretch of lysine residues, is highly basic, and can utilize heparan sulfate as its receptor. Nonetheless, the interaction between PTD_tat _and heparan sulfate is not only based on ionic intereactions, but also on the specific structures of the peptide and the proteoglycans [27-29]. Therefore, the choice of an infectivity-enhanced vector needs to be determined for each specific application involving gene delivery enhancement.

## Conclusion

Our data showed that a genetically modified Ad5 vector, Ad5.PTD_tat_, maintained the ability to interact with its native receptor CAR, and delivered transgenes into both high-CAR and low-CAR cells more efficiently than the unmodified Ad5 vector. Our data further showed Ad5.PTD_tat _infected cells via both CAR and PTD_tat _pathways. More significantly, Ad5.PTD_tat _exhibited enhanced gene delivery *in vivo *in a tumor model, and thus may be useful for gene therapy applications involving low gene delivery efficacy.

## Methods

### Cell culture

The human embryonic kidney 293 cells stably transformed with Ad-E1 DNA, human lung carcinoma A549 cells, human cervix adenocarcinoma Hela cells, human embryonic rhabdomyosarcoma RD cells, and human glioma D65MG and U118MG cells were all obtained from the American Type Culture Collection (ATCC, Manassas, VA). The 293 cells, A549 cells and U118MG cells were cultured in Dulbecco's modified Eagle's medium/Ham's F12 medium (DMEM/F12) containing 10% fetal bovine serum (FBS) and 2 mM L-glutamine. Hela cells were cultured cultured in minimum essential Eagle medium (MEM) containing 10% FBS and 2 mM L-glutamine. Both RD and D65MG cells were cultured in DMEM containing 10% FBS and 2 mM L-glutamine. All of the cells were maintained at 37°C in a 5% CO_2 _humidified incubator.

### Generation of the Ad5.PTD_tat _vector

Genetic modification of the Ad5 vector with PTD_tat _was achieved using our previously established fiber modification system [15]. In brief, the fiber shuttle vector containing a unique SnaBI restriction site immediately in front of the stop code of the fiber gene, named pNEB.PK.SnaBI, was used to generate a PTD_tat _modification. The sense and antisense oligonucleotides encoding the PTD_tat _motif, 5'-phos-ACT TTT TCA TAC ATT GCG CAA GAA GGC GGT GGA GGG TAT GGC AGG AAG AAG CGG AGA CAG CGA CGA AGA TAA TAA A-3' (sense) and 5'-phos-TTT ATT ATC TTC GTC GCT GTC TCC GCT TCT TCC TGC CAT ACC CTC CAC CGC CTT CTT GCG CAA TGT ATG AAA AAG T -3' (antisense), were annealed and cloned into the fiber shuttle vector pNEB.PK.SnaBI. This resulted in the fiber modified shuttle vector pNEB.PK.PTD_tat_. In order to incorporate the modified fiber into an Ad5 genome, pNEB.PK.PTD_tat _was linearized and recombined in *Escherichia coli *(E. *coli*) BJ5183 with a linearized Ad5 backbone plasamid pVK50 that contained the CMV promoter driven GFP reporter gene in its E1 region. After the positive recombinant plasmid, designated pAd5.PTD_tat_, was identified, stable and high quality plasmid was obtained from E. *coli *DH5α after re-transformation of the construct. The modification was confirmed by sequencing analysis.

The modified virus Ad5.PTD_tat _was rescued and purified as previously described [53]. In brief, the pAd5.PTD_tat _plasmid was digested with PacI (to release the viral genome), purified, and transfected into 293 cells stably expressing the complementary E1 genes. After the virus plaques formed, they were amplified in 293 cells, and purified utilizing a standard CsCl gradient protocol. The viral particle (VP) titer was determined using a conversion factor of 1.1 × 10^12 ^VPs/ml per absorbance unit at 260 nm.

### ELISA

The ELISA binding assay was performed essentially as described [15]. In brief, 10^9 ^VPs of either Ad5 or Ad5.PTD_tat _in 100 μl of 100 mM carbonate buffer (pH 9.5) was immobilized in each well of a 96-well maxisorp plate (Nunc, Roskilde, Denmark) by overnight incubation at 4°C. Following extensive washes with Tris-buffered saline (TBS) containing 0.05% Tween 20 (TBS-Tween), and blocking with 2% bovine serum albumin (BSA) in TBS-Tween, the viruses were incubated with varying amounts of purified recombinant sCAR. The binding of sCAR to the viruses was detected by incubation with anti-CAR antibody (Santa Cruz Biotechnology Inc., Santa Cruz, CA), followed by an AP-conjugated secondary antibody incubation. AP activity reflecting the amount of bound sCAR was determined using a color reaction with p-nitrophenyl phosphate (Sigma, St. Louis, MO) as recommended by the manufacturer. The absorbance at 405 nm (OD405) was obtained using PowerWaveHT 340 microplate reader (BioTek Instruments Inc., Winooski, VT).

### Cell binding assay

Cells were cultured in 6-well plates until they were confluent. The plate was then cooled down on ice, and incubated with Ad5 or Ad5.PTD_tat _at an MOI of 5000 VPs/cell for one hour at 4°C. After washing cells twice with cold phosphate buffered saline (PBS) on ice, the cells were collected by incubation with Versene (0.53 mM EDTA). After two more washes with PBS, the cells were lysed and processed to isolate DNA (Qiagen Inc., Valencia, CA). The viral copy number in the DNA samples were obtained by quantitative PCR using primers designed for the E4 region of adenoviral genome. The data were normalized against actin DNA in each sample.

### Gene transfer assay

Gene transfer efficacy of the viral vectors was assessed with the use of GFP reporter. In the assay, cells were plated in 24-well plates with a density of 10^5 ^cells per well the day before infection. Then the cells were infected with Ad5 or Ad5.PTD_tat _at MOIs of 100 or 500 VPs/cell as described previously [53]. Two days later, GFP expression was examined by fluorescence microscopy and quantified by a Synergy HT fluorescence plate reader (BioTek Instruments Inc., Winooski, VT).

### Competitive inhibition assays

Low-CAR U118MG cells or high-CAR A549 cells were plated in 24-well plates at a density of 10^5 ^cells per well the day before infection. Viruses equivalent to an MOI of 100 VPs/cell were used for each infection. To block cell surface CAR, recombinant knob protein was pre-incubated with cells at a final concentration of 50 μg/ml prior to viral infection [54], and to block the PTD_tat _epitope, the viruses were pre-incubated with 100 μg/ml of heparin [15,54]. Two hours after infection, the cells were washed with PBS, and refreshed with complete media containing 10% FBS. The cells were cultured for two days in the humidified 37°C, 5% CO_2 _incubator, and GFP microscopy was performed to examine the transgene expression.

### *In vivo *gene delivery

The subcutaneous tumors were established in athymic nude mice using 1 × 10^7 ^U118MG cells per tumor per mouse. After the tumors developed to ~0.5 cm in diameter, PBS or 10^10 ^VPs of Ad5 or Ad.PTD_tat _were injected into each tumor (n = 6). GFP expression was analyzed at 3, 7, and 10 days post infection using a custom-built non-invasive optical imaging system described previously [55]. The mice were placed in the imaging chamber under anesthesia with 3% isoflurane. Green fluorescence images were acquired at f/8 with 20-second exposure using a combination of excitation filter HQ487/15× and emission filter D535/30m (Chroma Technology, Rockingham, VT) supported by WinView32 software (Roper Scientific Inc., Trenton, NJ). All of the procedures involving animals were approved by the Institutional Animal Care and Use Committee of the University of Alabama at Birmingham and performed according to their guidelines.

## Competing interests

The author(s) declare that they have no competing interests.

## Authors' contributions

TH participated in the generation and *in vitro *characterization of the adenoviral vectors. YT carried out *in vitro *and *in vivo *gene transfer assays. HU performed immunohistochemistry studies. LEP participated in cell culture and tumor model establishment. GPS helped in immunohistochemical studies and in the preparation of the manuscript. JLC assisted in the design of the study and manuscript preparation. HW conceived of the study, participated in its design and coordination, and drafted the manuscript. All authors read and approved the final manuscript.
